# Methylated BSA Mimics Amyloid-Related Proteins and Triggers Inflammation

**DOI:** 10.1371/journal.pone.0063214

**Published:** 2013-05-01

**Authors:** Jeremy Di Domizio, Stephanie Dorta-Estremera, Wei Cao

**Affiliations:** Department of Immunology, University of Texas M. D. Anderson Cancer Center, Houston, Texas, United States of America; University of São Paulo, Brazil

## Abstract

The mechanistic study of inflammatory or autoimmune diseases requires the generation of mouse models that reproduce the alterations in immune responses observed in patients. Methylated bovine serum albumin (mBSA) has been widely used to induce antigen-specific inflammation in targeted organs or in combination with single stranded DNA (ssDNA) to generate anti-nucleic acids antibodies in vivo. However, the mechanism by which this modified protein triggers inflammation is poorly understood. By analyzing the biochemical properties of mBSA, we found that mBSA exhibits features of an intermediate of protein misfolding pathway. mBSA readily interact with a list of dyes that have binding specificity towards amyloid fibrils. Intriguingly, mBSA displayed cytotoxic activity and its binding to ssDNA further enhanced formation of beta-sheet rich amyloid fibrils. Moreover, mBSA is recognized by the serum amyloid P, a protein unanimously associated with amyloid plaques in vivo. In macrophages, we observed that mBSA disrupted the lysosomal compartment, signaled along the NLRP3 inflammasome pathway, and activated caspase 1, which led to the production of IL-1β. In vivo, mBSA triggered rapid and prominent immune cell infiltration that is dependent on IL-1β induction. Taken together, these data demonstrate that by mimicking amyloidogenic proteins mBSA exhibits strong innate immune functions and serves as a potent adjuvant. These findings advance our understanding on the underlying mechanism of how aberrant immune responses lead to autoimmune reactions.

## Introduction

Antigen-induced arthritis has been widely studied in animals as a model of rheumatoid arthritis. This chronic inflammation of the joints can be induced by immunization of animals with an antigen and intraarticular re-challenge with the same antigen in the presence of complete Freund’s adjuvant several days later [1]. Methylated bovine serum albumin (mBSA) is by far the most effective antigen at inducing prolonged inflammation in different strains of rabbits, mice, and other rodents [2]. The addition of positive electrical charges by methylation of the anionic native BSA has been speculated as the determinant factor in the chronicity of the inflammation induced [3]. In particular, the cationic mBSA was found retained for a longer time in articular connective tissues than negatively charged antigens, leading to a delayed release of antigens and favoring the in situ immune complex formation and deposition [3].

Interestingly, mBSA has also been used as a carrier protein for the induction of anti-DNA antibodies in other autoimmune disease models [4]–[9]. DNA is poorly immunogenic by itself and immunization of mice with nucleic acids fails to induce detectable anti-DNA titers. However, mice receiving denatured single stranded DNA from different sources complexed to mBSA develop anti-DNA antibodies approximating the serology observed in systemic lupus erythematosus (SLE) patients [4], [10]. In these models, it was thought that the increased adjuvancy of mBSA is responsible for breaking tolerance to nucleic acids, yet the precise mechanism by which it is achieved is unknown. Nevertheless, mBSA was included as a carrier protein to obtain high titer antibodies in other studies [11], [12].

Adjuvants are substances that are included in vaccines to critically enhance the magnitude and modulate the quality of the protective immune responses. Not until recently, the mechanism how adjuvants fulfill such function has been revealed. Among different types of adjuvants used in clinics or in experimental animals, they universally show strong capacity to trigger inflammation and activate different aspects of the innate immune system, which prime the adaptive immune system to induce antibody or cellular responses. In particular, the oil-based aluminum adjuvant has been shown to exert a direct effect on inflammasome action and IL-1β production, a key mediator of inflammation. The application of adjuvants and their principle of action are not limited to vaccines to prevent infectious diseases, but are increasingly tested in cancer immunotherapy, where anti-tumor specific response is intentionally induced.

Amyloid fibrils are stable insoluble aggregates of terminal misfolded protein products with extensive beta sheet structures [13]–[15]. These misfolded particulates participate in inflammatory responses in both central nervous system and peripheral organs, mainly by activating inflammasome and inducing IL-1β secretion [16]–[19]. Recently we observed that the precursor form of amyloid, soluble protein oligomers, can efficiently bind DNA converting them into amyloids [20]. Strikingly, these nucleic acid-containing amyloids could initiate inflammation and their injection to non-autoimmune mice lead to a broad anti-autoantigen response with the generation of anti-DNA autoantibodies [21].

Here we show that mBSA shares properties with both oligomeric amyloid precursors and fibrous amyloid. mBSA is able to activate the inflammasome in macrophages and induce profound immune cell infiltration in vivo. Thus the amyloidogenic properties of mBSA render it capable to initiate inflammation and may explain the adjuvant effect observed in vivo.

## Materials and Methods

### Reagents

Bovine serum albumin (BSA), mBSA and thioglycollate medium were purchased from Sigma. Amyloidogenic peptides were obtained commercially: Aβ (1–42) from EMD Biosciences and reverse Aβ peptide from California Peptide Research.

### Binding with Amyloid-specific Dyes

The assays were performed as described previously [20]. Briefly, BSA samples (0.5 mg/ml) were mixed with 50 µM of Thioflavin T (Sigma-Aldrich) and measured for fluorescence emission in a spectrofluorometer (Jasco FP-6500) with an excitation wavelength of 450 nm and an emission between 450–600 nm. Proteins were also mixed with 5 µM of 4,4-dianilino-1,1-binaphthyl-5,5-disulfonic acid dipotassium salt (bis-ANS; Sigma-Aldrich). The fluorescence was measured in spectrofluorometer with an excitation wavelength of 395 nm and an emission between 420 and 580 nm. Alternatively, BSA samples (50 µg/ml) were pre-mixed with denatured salmon sperm ssDNA (50 µg/ml) for 2 hours at room temperature. After incubating with Congo Red (30 µg/ml) for 30 min, the fluorescence was measured in a spectrofluorometer with an excitation wavelength of 497 nm and an emission between 600 and 700 nm. For microscopic analysis, BSA samples (500 µg/ml) were first mixed with denatured salmon sperm ssDNA (500 µg/ml) for 2 hours at room temperature. Samples were then deposited in wells of a positive charged Teflon printed slide, 8 well 6 mm diameter (Electron Microscopy Sciences) and air dried. Samples were fixed in 4% paraformaldehyde, washed in water and stained with 1% Thioflavin S or 1% Congo Red in 80% Ethanol, 100 mM NaOH. Slide was washed in 80% Ethanol, air dried, and analyzed microscopically in bright and polarized light using Olympus BX41 microscope.

### Transmission Electron Microscopy Analysis

Samples were placed on 400 mesh carbon coated, formvar coated nickel grids treated with poly-l-lysine for 1 hour. Excess samples were blotted with filter paper, then stained with filtered aqueous 2% uranyl acetate for 2 mins. Stain was blotted dry from the grids with filter paper and samples were allowed to dry. Samples were then examined in a JEM 1010 transmission electron microscope (JEOL, USA, Inc., Peabody, MA) at an accelerating voltage of 80 Kv. Digital images were obtained using the AMT Imaging System (Advanced Microscopy Techniques Corp., Danvers, MA).

### Nucleic Acid-binding Gel Shift Assay

Different proteins were incubated with 0.5 µg of denatured salmon sperm ssDNA in TE buffer (10 mM Tris-Cl, pH 7.4 and 1 mM EDTA) for 60 min. The samples were then loaded onto 1% agarose gel and subjected to electrophoresis separation. For inhibition with glycosaminoglycan, BSA proteins were pre-incubated with different amounts of heparin for 15 min prior to mixing with DNA.

### Binding of SAP to mBSA

Different concentrations of Aβ peptide (1-42), Aβ (42-1), mBSA or mBSA+ssDNA in carbonate buffer were coated in ELISA plates overnight at 4°C. The plates were blocked with buffer containing 150 mM NaCl, 25 mM Tris, 1% BSA, 2 mM CaCl_2_ for 1 hour at room temperature. After several washes, biotinylated human SAP (Calbiochem) (4 µg/ml) was added for 2 hours. Then, streptavidin-HRP was added for 1 hour and TMB used as the substrate. Absorbance at 450 nm was read using a spectrophotometer SpectraMax M5e (Molecular Devices).

### Cytotoxicity

Proteins were incubated with RPMI 8226 (1×10^6^/ml) at different concentrations in culture medium at 37°C for 24 hrs. Cells were washed, resuspended in propidium iodide containing buffer (BD Bioscience) and analyzed on a *FACSCalibur™* cytometer (BD Bioscience). In some experiments, proteins were mixed with ssDNA or heparin at 1∶1 ratio 30 min prior to adding to the cells.

### Preparation and Stimulation Bone-marrow Derived Macrophages

Bone marrows of 8- to 12-wk-old Balb/c mice were harvested and cells were cultured in RPMI medium +10% FBS supplemented by 20% L929 supernatants for 5 days to differentiate macrophages. BMDMs at 5×10^5^ cells/ml were primed with 10 ng/ml LPS for 4 hours then stimulated with 5 µg/ml BSA/mBSA ±5 µg/ml ssDNA or 10 µM Aβ/reverse peptides for 2 hours. Supernatants were collected to measure IL-1β secretion by ELISA. In some experiments, LPS-primed cells were pre-incubated with 10 µg/ml Cytochalasin B, 50 mM KCL, 25 µg/ml Glybenclamide or 50 µM Ac-YVAD-CMK 15 min before stimulation. To analyze lysosomes integrity, BMDM cells were stained with 1 µg/ml acridine orange and then stimulated as previously for 3 hours. For the caspase 1 analysis, stimulated cells were stained with the FAM-YVAD-FMK FLICA™ Caspase 1 reagents (Immunchemistry Tech.) according to the manufacturer’s protocol.

### Mice and Injections

Wild-type BALB/cByJ mice were obtained from The Jackson Laboratory. All experiments with mice were performed in compliance with the principles and procedures outlined in the NIH Guide for the Care and Use of Animals and were approved by The University of Texas MD Anderson Cancer Center Animal Care and Use Committee. To evaluate innate immune response, the 8- to 12-wk-old Balb/c mice received *i.p.* 30 µg of endotoxin-free denatured ssDNA (Invivogen) together with 75 µg of BSA or mBSA in 200 µl PBS or 1 ml of 3% TG. In other experiments, 8- to 12-wk-old C57BL/6J (The Jackson Laboratory) and IL-1β^−/−^ mice (kindly provided by Dr. Yoichiro Iwakura) were used. Female and male mice were equally divided among the test groups and analyzed.

### Analysis of Peritoneal Inflammation

Peritoneal exudate cells were harvested at 4 hours after injection of stimuli. FACS analysis was performed with fluorophore-conjugated Ab against F4/80 (Biolegend), Ly6C, Ly6G (Miltenyi), and CD11b (BD Bioscience) then analyzed on an LSR II. Countbright absolute counting beads (Molecular Probes) were used to calculate cell numbers. Cell populations were qualified as CD11b^+^ Ly6C^+^ F4/80^high^ macrophages, CD11b^+^ Ly6G^−^ Ly6C^+^ monocytes and CD11b^+^ Ly6C^−^ Ly6G^+^ neutrophils. Quantitative PCR analysis on peritoneal exudate cells were performed with the primers listed in Table 1.

**Table 1:** Sequences of primers used for quantitative PCR analysis.

Gene	Forward	Reverse
s18	CCATTCGAACGTCTGCCCTAT	GTCACCCGTGGTCACCATG
il-1α	TTGGTTAAATGACCTGCAACA	GAGCGCTCACGAACAGTTG
il-1β	TGTAATGAAAGACGGCACACC	TCTTCTTTGGGTATTGCTTGG
il-18	CAAACCTTCCAAATCACTTCCT	TCCTTGAAGTTGACGCAAGA
il-1rn	GGCAGTGGAAGACCTTGTGT	CATCTTGCAGGGTCTTTTCC
il-6	GCTACCAAACTGGATATAATCAGGA	CCAGGTAGCTATGGTACTCCAGAA
il-10	CAGAGCCACATGCTCCTAGA	TGTCCAGCTGGTCCTTTGTT
il-12p40	CCATCAGCAGATCATTCTAGACAA	CGCCATTATGATTCAGAGACTG
il-23a	TCCCTACTAGGACTCAGCCAAC	TGGGCATCTGTTGGGTCT
ifnγ	AACTGGCAAAAGGATGGT	GACCTCAAACTTGGCAATAC
ccl2	CATCCACGTGTTGGCTCA	GATCATCTTGCTGGTGAATGAGT
ccl3	TGCCCTTGCTGTTCTTCTCT	GTGGAATCTTCCGGCTGTAG
ccl4	GCCCTCTCTCTCCTCTTGCT	GAGGGTCAGAGCCCATTG
ccl5	TGCAGAGGACTCTGAGACAGC	GAGTGGTGTCCGAGCCATA
ccl11	AGAGCTCCACAGCGCTTCT	GCAGGAAGTTGGGATGGAG
ccl12	CCATCAGTCCTCAGGTATTGG	CTTCCGGACGTGAATCTTCT
cxcl1	TGACAGCGCAGCTCATTG	AGACTCCAGCCACACTCCAA
cxcl9	CTTTTCCTCTTGGGCATCAT	GCATCGTGCATTCCTTATCA
cxcl11	GCTGCTGAGATGAACAGGAA	CCCTGTTTGAACATAAGGAAGC
cxcl12	CTGTGCCCTTCAGATTGTTG	TAATTTCGGGTCAATGCACA

### Statistical Analysis

Statistical analyses were performed with the two-tailed unpaired Student t-test or with a two-way ANOVA test with Bonferroni post-test using GraphPad Prism 5.0 software. P>0.05 was considered nonsignificant.

## Results

### mBSA Exhibits Biochemical Features Related to Amyloids

**Figure 1:**
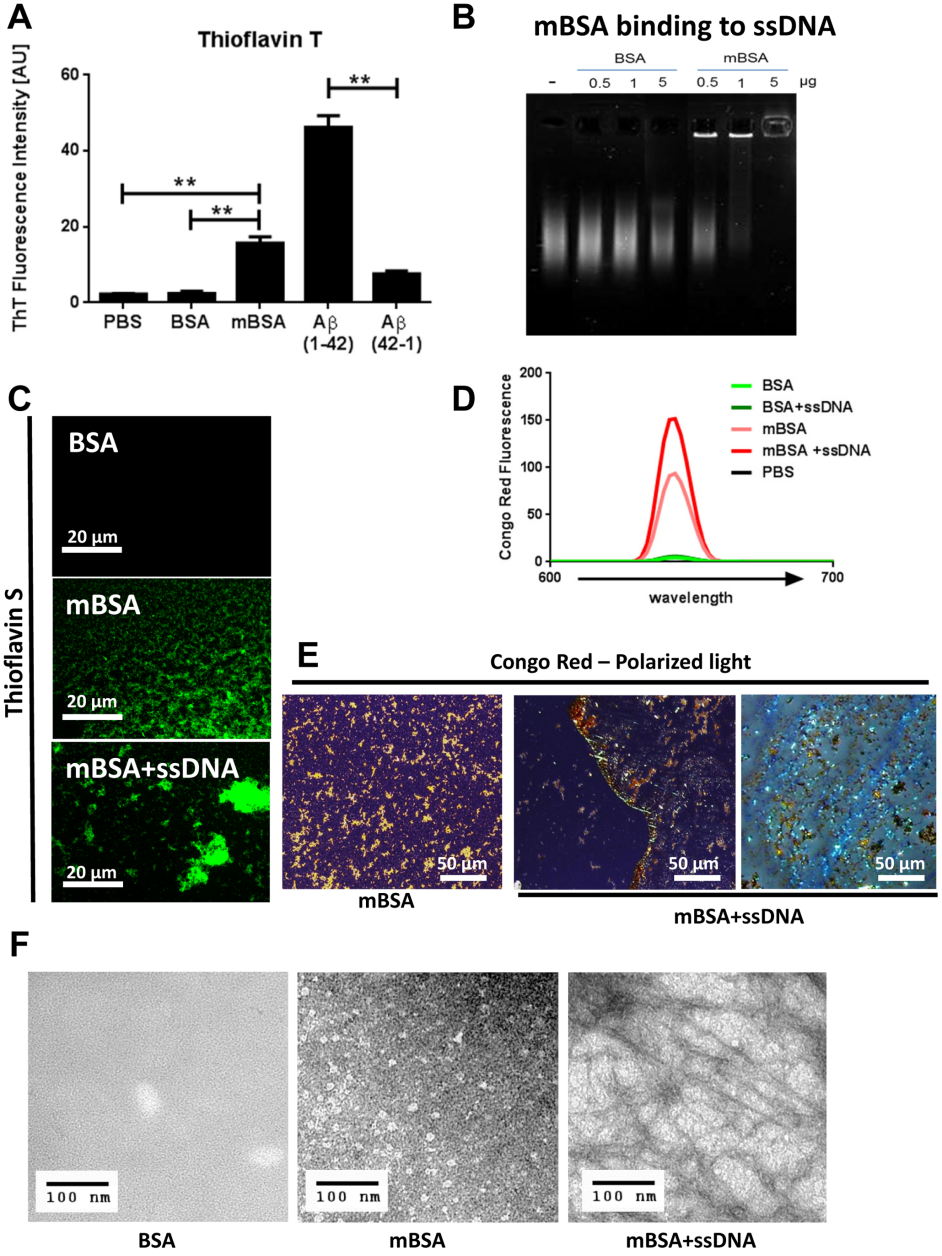
mBSA displays features of amyloid. (A) Fluorescence intensity of Thioflavin T at 480 nm in buffer, with BSA, mBSA, Aβ or with the reverse control peptide. Error bars are means ± SD of 3 independent experiments. **p<0.005. (B) Gel shift analysis of salmon sperm ssDNA pre-mixed with different amounts of BSA or mBSA. (C) Thioflavin S fluorescence in the presence of different BSA proteins or aggregates observed under fluorescence microscopy. Original magnification 100X. scale bar: 20 µm. (D) Fluorescence emission profiles of Congo Red in the presence of buffer, different proteins or aggregates. (E) Birefringence of Congo Red on mBSA or mBSA plus ssDNA aggregate observed by polarized light microscopy. Original magnification 40X. bar: 50 µm. (F) Transmission electron microscopy analysis of mBSA or mBSA plus ssDNA. Bar: 100 nm.

Amyloids are insoluble fibrous protein aggregates that display a cross-beta sheet quaternary structure that can be recognized by planar aromatic dyes like Thioflavin or Congo Red [22], [23]. Surprisingly, dye Thioflavin T showed an increased fluorescence emission in the presence of mBSA but not in the presence of the native BSA, suggesting that the methylation of the protein led to the formation of beta sheet rich structures (Fig. 1A). Nucleic acids are negatively charged molecules, and bind efficiently to cationic groups. Whereas native BSA does not bind to denatured ssDNA, mBSA can form complexes with ssDNA in a dose dependent manner (Fig. 1B). We then wondered whether such interaction would change the biochemical properties of mBSA. Since Thioflavin T reacts with nucleic acids [24], we used a closely related dye Thioflavin S, which also emitted fluorescence upon binding to mBSA (Fig. 1C). Interestingly, mBSA mixed with ssDNA resulted in highly aggregated complexes that are positive with Thioflavin S (Fig. 1C).

In contrast to native BSA, mBSA has limited solubility in water and frequently precipitates on slides, suggesting methylation has tremendous impact on the biophysical property of the protein. To further analyze the beta sheet content in mBSA, we tested Congo red, a dye specific for amyloid [22], [23]. Whereas no fluorescence of the dye could be observed in the presence of BSA or BSA plus ssDNA, the Congo red fluorescence was highly detectable from mBSA (Fig. 1D). Furthermore, binding with ssDNA significantly enhanced Congo red emission. When observed under polarized light, Congo red staining on mBSA emitted yellow-orange glow, whereas mBSA+ssDNA complexes gave an apple-green birefringence, characteristic of amyloid containing cross-beta sheets (Fig. 1E). To further confirm this finding, we analyzed the samples by transmission electron microscopy and detected fibrous structures highly abundant within the mBSA+ssDNA complexes (Fig. 1F). mBSA, in contrast to native BSA, formed non-fibrous particulates under the same condition. Therefore through complexing with DNA, mBSA displays classic characteristics of beta sheet rich amyloid.

### mBSA Display Properties of Protein Misfolding Intermediate

**Figure 2:**
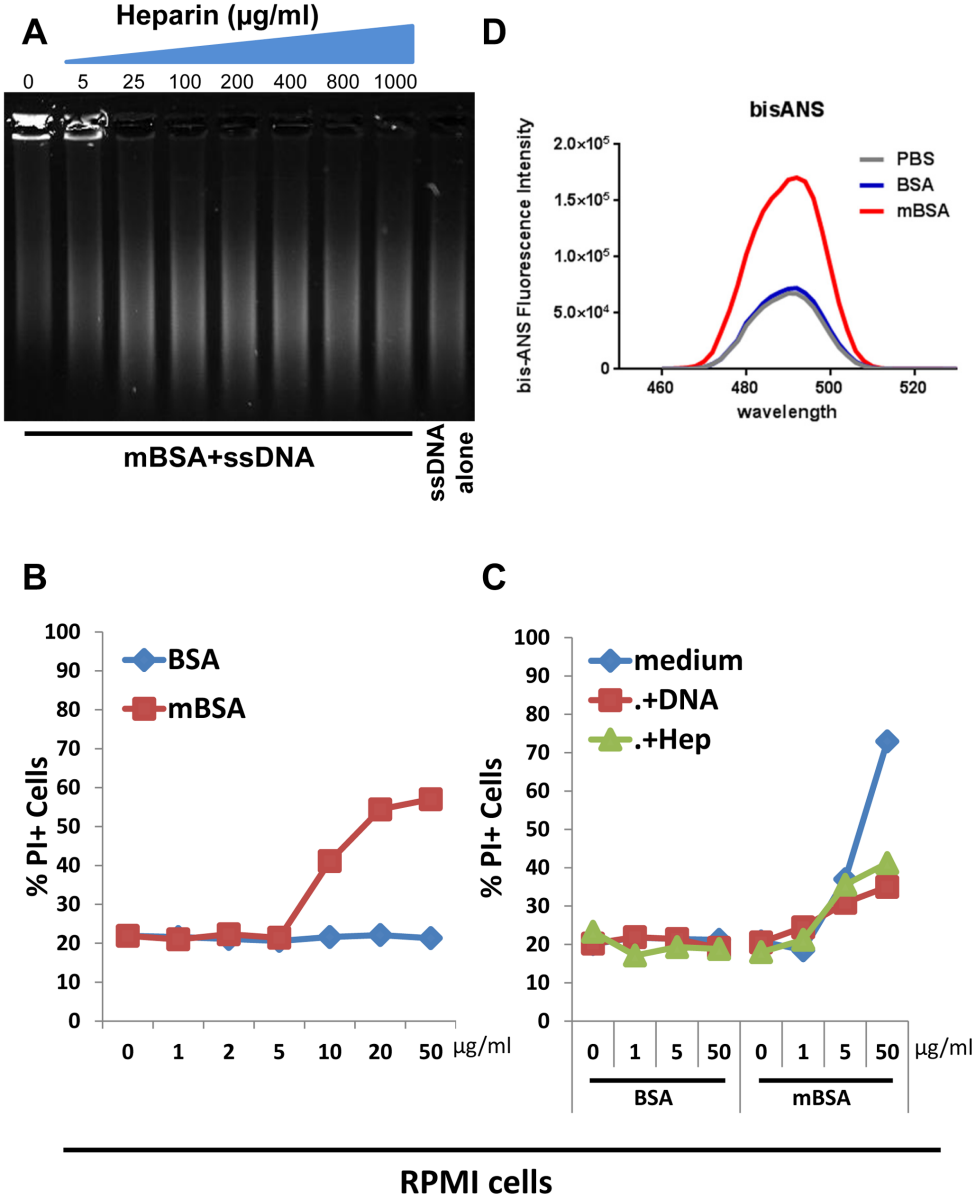
mBSA shares properties with soluble amyloid precursor. (A) Gel shift analysis of salmon sperm ssDNA that was pre-mixed with mBSA in the presence of different amounts of heparin. (B) Assessment of cell death of RPMI 8226 cells cultured 24 hrs with different amounts of BSA or mBSA. Shown are representative results of PI staining from 2 independent experiments. (C) Assessment of dead RPMI 8226 cell population cultured with different amounts of BSA or mBSA that were pre-incubated with medium, DNA or heparin. Results shown are representative of 2 independent experiments. (D) Fluorescence emission profiles of bis-ANS obtained after incubation in PBS, with BSA or with mBSA.

The formation of insoluble amyloid requires the generation of soluble oligomeric proteins, which are precursors of amyloids with unusual cytotoxicity [25], [26]. We previously demonstrated that binding with nucleic acids or glycosaminoglycans converts soluble protein oligomers into insoluble inert amyloids [20]. Having observed that mBSA interacts with DNA and gains enhanced Congo red fluorescence made us hypothesize that mBSA may share certain features with amyloid precursors. Analogous to stabilized protein oligomer [20], binding of mBSA to DNA was inhibited by the presence of increasing concentration of heparin, a prototypical glycosaminoglycan (Fig. 2A). Soluble protein oligomers, but not the insoluble amyloid fibrils, have the capacity to disrupt the lipid bilayer of cell membranes to cause cell death [27]. Interestingly, mBSA, but not BSA, displayed cytotoxicity towards a human plasmacytoma line RPMI 8226 cells in a dose dependent manner (Fig. 2B). Similar to stabilized protein oligomer [20], such cytotoxicity could be abolished in the presence of ssDNA or heparin (Fig. 2C).

To understand the molecular basis of such intriguing cellular effect, we examined further the biochemical properties of mBSA. As an intermediate of protein misfolding, soluble protein oligomers display structural alteration that is distinct from the native form of the proteins [28], [29]. mBSA, but not the native form, gave rise to prominent fluorescent emission profiles with 4,4-bis (1-anilinonaphthalene 8-sulfonate) (bis-ANS) (Fig. 2D), a fluorescent dye preferentially binds to hydrophobic areas of a protein structure [30]. This indicates the presence of exposed hydrophobic regions that are normally buried in natively folded structures, consistent with previous reports on soluble protein oligomers [20], [28], [29].

**Figure 3:**
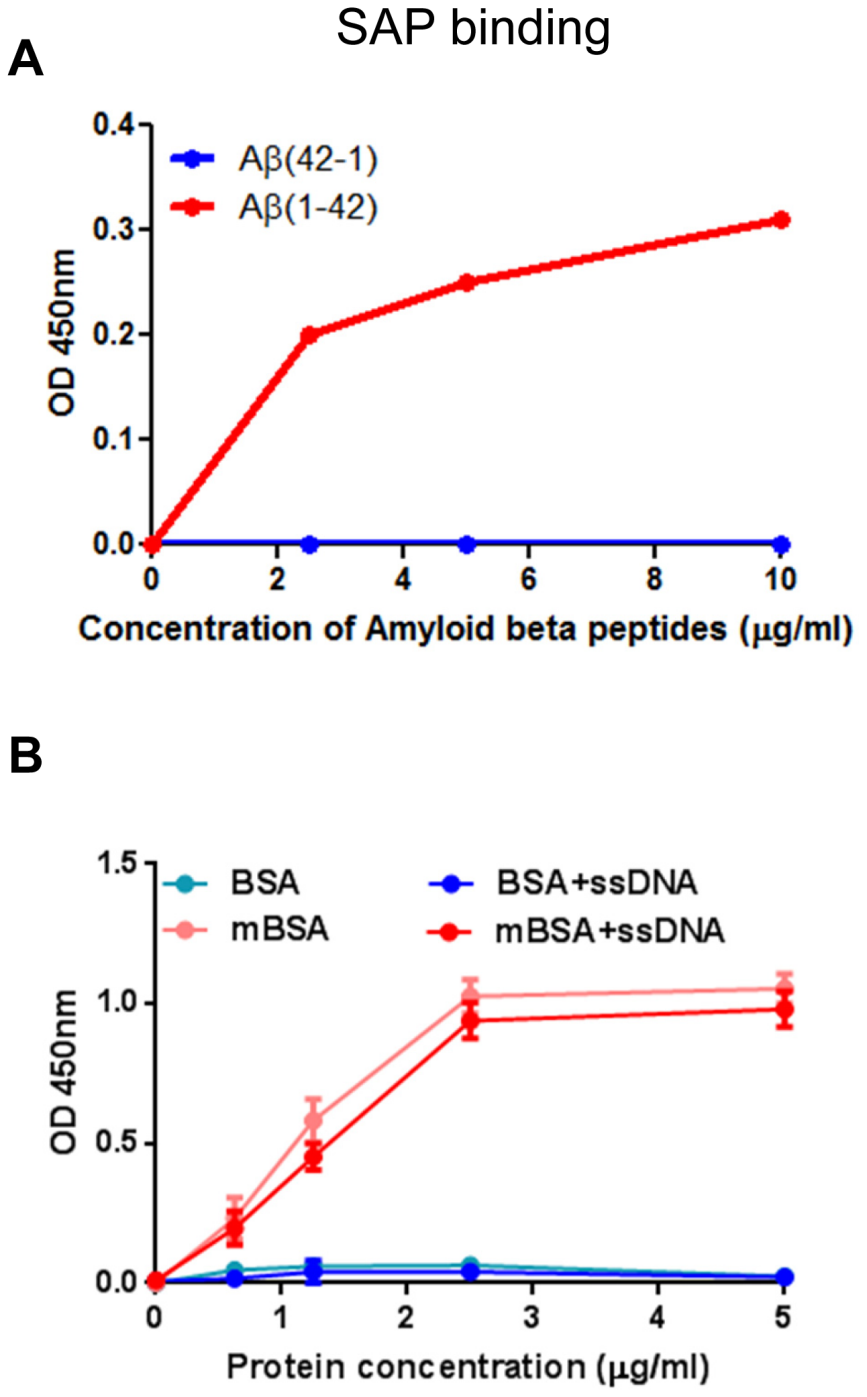
Serum amyloid P binds to mBSA. (A) Binding of SAP to different amounts of Aβ or the reverse control peptide was assessed by ELISA. Similar results were obtained from 2 independent experiments. (B) Binding of SAP to different amounts of BSA or mBSA with or without ssDNA was assessed by ELISA. Error bars are means ± SD of 2 independent experiments.

Serum amyloid P (SAP) is a member of pentraxins that specifically associates with beta sheet rich amyloid fibrils [31]–[33]. In vitro, SAP binds to amyloidogenic Amyloid β (Aβ) peptide, but not to a control peptide with reversed sequence (Fig. 3A). Interestingly, SAP positively recognizes both mBSA and mBSA+ssDNA, but not BSA nor DNA, in a similar dose-dependent manner (Fig. 3B). Altogether, these results suggest that mBSA represents an intermediate of protein misfolding pathway that can lead to the formation of amyloid.

### mBSA Triggers IL-1β Production by Activating Inflammasome

**Figure 4:**
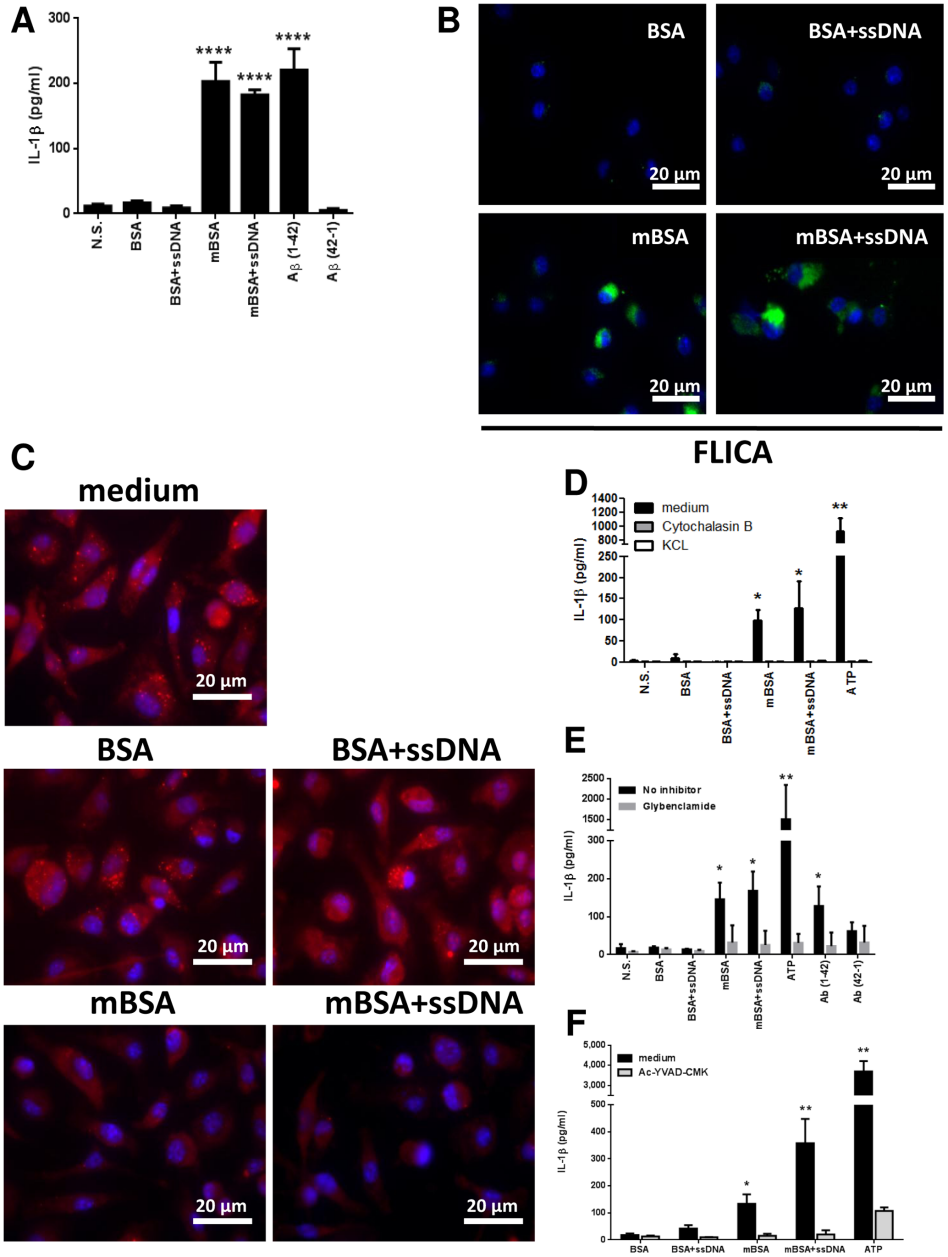
mBSA elicits IL-1β production by activating inflammasome. (A) Secretion of IL-1β by BMDM stimulated with different forms of BSA or Aβ peptides for 2 hours. Error bars are means ± SD of 5 independent experiments. ****p<0.0001. (B) Caspase 1 activation in BMDMs stimulated with different forms of BSA analyzed by FLICA assay. Representative result of 3 independent experiments is shown. Original magnification 100X. bar: 20 µm. (C) Acridine orange staining of BMDM stimulated by different forms of BSA to reveal lysosome integrity. Original magnification of fluorescence microscopy 100X. bar: 20 µm. (D) Secretion of IL-1β by BMDM induced by different forms of BSA in the presence of different inhibitors. Error bars are means ± SD of 2 independent experiments. *p<0.05, **p<0.005. (E) Secretion of IL-1β by BMDM induced by different forms of BSA or Aβ peptides in the presence or not of the NRLP3 inhibitor glybenclamide. Error bars are means ± SD of data obtained with cells from 4 different mice. *p<0.05, **p<0.005. (F) Secretion of IL-1β by BMDM induced by different forms of BSA in the presence of the caspase 1 inhibitor Ac-YVAD-CMK. Error bars are means ± SD of data obtained with cells from 3 different mice. *p<0.05, **p<0.005.

It has been demonstrated that amyloidogenic peptides Aβ can activate the NLRP3 inflammasome in bone marrow-derived macrophages (BMDMs), which leads to the production of IL-1β [17], [19]. In addition, oligomers of islet amyloid polypeptide (IAPP) were shown to similarly activate NLRP3 inflammasome [18]. We then assessed the capacity of mBSA and mBSA+ssDNA to similarly stimulate BMDMs. First of all, neither of them directly induced pro-IL-1β or IL-1β expression in BMDMs. As TLR/NF-κB pathway is potent at inducing pro-IL-1β expression, inflammasome activation then leads to maturation and release of IL-1β. Interestingly, both mBSA and mBSA+ssDNA induced LPS-primed BMDMs to secrete IL-1β to a similar extend as Aβ peptides (Fig. 4A). None of the control proteins, BSA or the reverse peptides of Aβ, induced significant IL-1β. To further dissect the mechanism by which the production of IL-1β was reached, we stained the stimulated cells with the fluorescent probe FAM-YVAD-FMK to label the active caspase 1, an enzyme that processes IL-1β maturation. As expected, BMDMs only when stimulated by mBSA or mBSA+ssDNA had increased fluorescence in their cytoplasm, suggesting that caspase 1 was activated by these stimuli (Fig. 4B).

Initiation of the inflammasome complex formation in macrophages largely depends on the activation of Cathepsin B following phagosomal destabilization [19], [34]. The integrity of the lysosomal compartment in cells can be verified by staining the cells with the acridine orange, a dye that accumulates in lysosomes. Strikingly, macrophages exposed to mBSA and mBSA+ssDNA showed a significantly decreased acridine orange staining compared to cells cultured with BSA controls, suggesting that both mBSA and mBSA+ssDNA triggered lysosomal disruption in macrophages (Fig. 4C). It is also known that activation of the inflammasome requires endocytosis of particulates and the induction of potassium efflux. In the presence of the endocytosis inhibitor cytochalasin B or increasing concentration of KCL, the IL-1β released by mBSA-stimulated BMDMs was drastically abolished, similarly to their effects on inflammasome activator ATP (Fig. 4D).

Since both Aβ and IAPP activate NLRP3 inflammasome to prompt IL-1β maturation [16], [19], we further examined the role of this signaling pathway in mediating mBSA-induced IL-1β. Similar to Aβ, both mBSA and mBSA+ssDNA are sensitive to the pre-treatment of glybenclamide, an inhibitor acts downstream of the P2X7 receptor but upsteam of NLRP3 [35], on BMDM when stimulating IL-1β release (Fig. 4E). Following the assembly of the NLRP3 inflammasome, caspase 1 is activated by autocatalytic processing that triggers the maturation of IL-1β and its release. As expected, the secretion of IL-1β induced by mBSA is abolished in the presence of the caspase 1 inhibitor Ac-YVAD-CMK (Fig. 4F). These results collectively demonstrate that mBSA is able to induce IL-1β secretion by likely signaling through the NLRP3 inflammasome pathway and activating caspase 1 complexes in macrophages.

### mBSA Elicits IL-1β-dependent Inflammation in vivo

**Figure 5:**
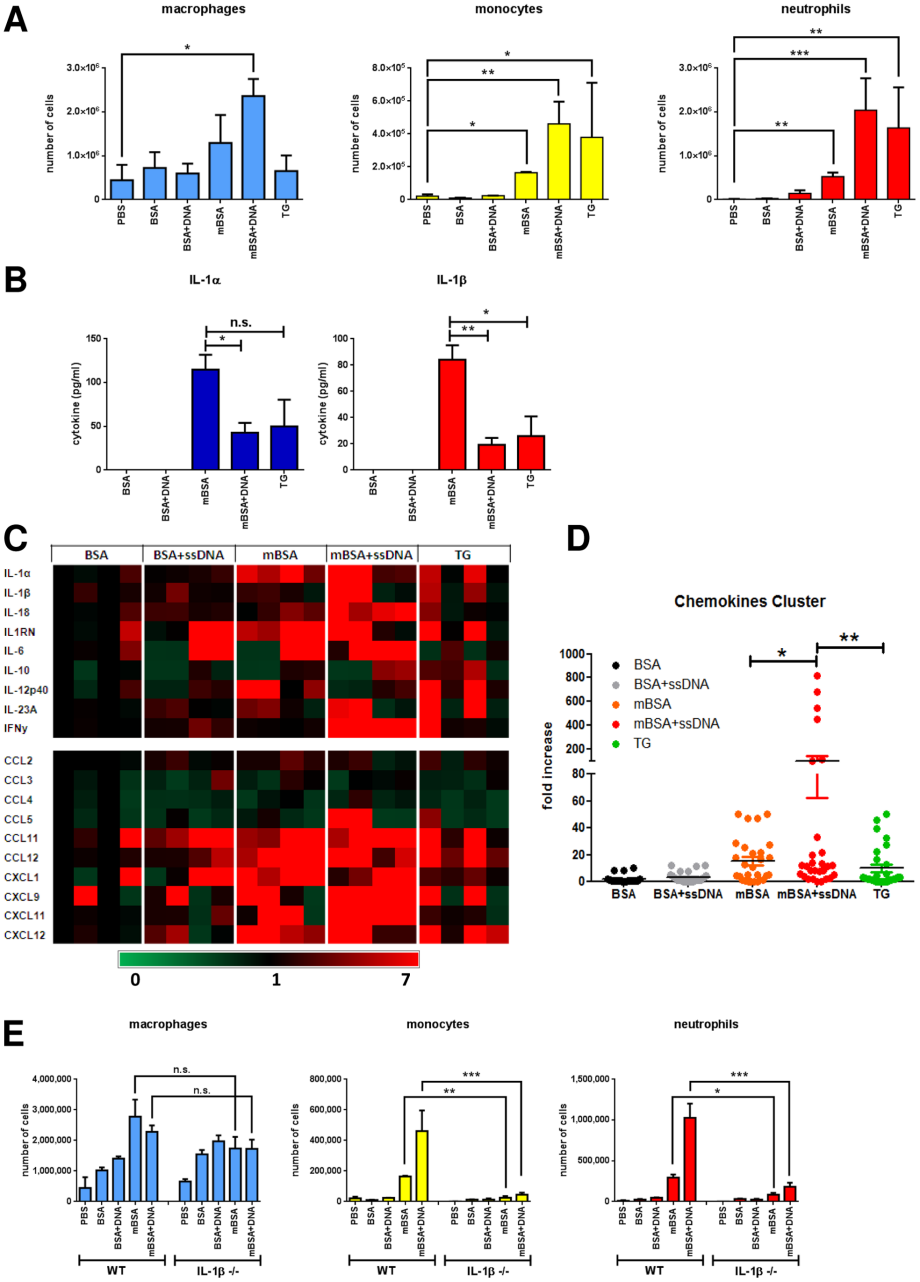
mBSA triggers inflammation in vivo. (A) Numbers of infiltrating macrophages (left), monocytes (middle) and neutrophils (right) in the peritoneum of mice 4 h after *i.p.* injection of different stimuli. Error bars are means ± SD of 4 mice per group. *p<0.05, **p<0.005. (B) Levels of IL-1α (left) and IL-1β (right) secreted in the peritoneal lavages. *p<0.05. (C) Gene expression of peritoneal exudate cells presented as a heat map. One BSA-injected animal was used as a reference. Each block represents one mouse. (D) Plot of induced transcript expression of the chemokines from the bottom cluster of *p<0.05, **p<0.005. (C). (E) Numbers of infiltrating macrophages (left), monocytes (middle) and neutrophils (right) in the peritoneum of wild-type or IL-1β^−/−^ mice 4 h after *i.p.* injection of different stimuli. Error bars are means ± SD of 3 mice per group. **p<0.005.

Stimulation of the inflammasome and release of IL-1β can be the initial steps in an inflammatory cascade, an important property of many adjuvants [36]. To demonstrate the innate immune function of mBSA, we injected mice intraperitoneally with BSA, mBSA or thioglycollate medium (TG). In comparison, we also inoculated endotoxin-free ssDNA together with either BSA or mBSA. Leukocytes infiltrating the peritoneal cavity 4 hours following injection were analyzed by FACS. Interestingly, mBSA induced significant recruitment of cells, especially with increased numbers of neutrophils and macrophages (Fig. 5A). Remarkably, mBSA plus ssDNA triggered the most abundant leukocyte infiltration, more than what induced by TG, a stimulatory agent routinely used to induce peritoneal inflammation for elicitation of macrophages.

Consistent with the result of BMDM stimulation, both mBSA and mBSA+ssDNA induced IL-1β secretion which was detectable from the peritoneal fluid (Fig. 5B). Secretion of IL-1α, a closely related cytokine that shares the receptor with IL-1β, was also prompted by mBSA, mBSA+ssDNA and TG, the stimuli that triggered peritoneal inflammation. Of note, mBSA was more potent at inducing IL-1α/β than mBSA+ssDNA or TG. Further analysis of the gene expression of the peritoneal exudate cells revealed that the transcripts of IL-1 family cytokines, including IL-1α, IL-1β and IL-18, were largely upregulated after inoculation of mBSA, mBSA+ssDNA and TG (Fig. 5C). Interestingly, mBSA+ssDNA selectively augmented the transcription of cytokine IFNγ. In addition, transcripts encoding chemokines, such as CCL5, CCL11, CCL12, CXCL1, CXCL9, CXCL11 and CXCL12, were all upregulated by mBSA, mBSA+ssDNA and TG. Opposite of weaker IL-1α/β induction, mBSA+ssDNA injection led to a significantly higher expression of this group of chemokines over mBSA (Fig. 5D).

To assess the role of IL-1β in mBSA and mBSA+ssDNA induced inflammation, we inoculated these molecules and corresponding controls in wild type or IL-1β−/− mice *i.p.* Interestingly, IL-1β deficiency significantly abolished the peritonitis induced by mBSA and mBSA+ssDNA (Fig. 5E). Thus, mBSA and its derived molecular complex can potently trigger inflammation via induction of IL-1β as a result of inflammasome activation.

## Discussion

In recent years, aberrantly folded proteins in the form of amyloid fibrils and misfolded protein aggregates have been recognized as a special class of danger signals that trigger inflammation via inflammasome activation to exacerbate diverse human diseases, such as Alzheimer’s disease and type 2 diabetes [18], [19], [37], [38]. The present study describes the biochemical and cellular mechanism underlying the unexpected innate immune function of mBSA, a modified BSA protein used in various in vivo disease models. In addition to altered charges, methylation significantly affects the folding of BSA molecule and generates an intermediate of protein misfolding pathway that allows the formation of amyloid. We have shown that a complex containing mBSA and ssDNA display characteristics of beta sheet rich amyloid. Consistent with previous reports on natural amyloids, mBSA and mBSA+ssDNA signal along the NLRP3 inflammasome pathway and induce maturation of IL-1β. In addition, these agents are potent to trigger IL-1β-dependent peritoneal inflammation when inoculated in vivo.

Our study strongly suggests that mBSA is not a normal protein antigen that triggers regular memory immune response when injected to tissues. It rather acts as a self-adjuvanted antigen to induce profound local inflammation while presenting the antigenic backbone. The capacity of mBSA to induce a sustained organ-specific inflammation has been thought to depend on its cationic property which delays its release in vivo. Yet, cationization of proteins is not sufficient per se to elicit an inflammatory response, since intra-articular injection of cationic myoglobulin or lysozyme had no effect [39]. Our results indicate that mBSA may contribute to local tissue inflammation at least partly by direct induction of IL-1 family cytokine production via inflammasome activation. In fact, Verri et al, reported the significant amount of IL-1β induced by mBSA in cutaneous and articular hypernociception and effective inhibition of hypernociception by treatment with IL-1ra [40]. Displaying properties of soluble protein oligomers, mBSA may also preferentially deposit onto the proteoglycans present in the extracellular matrix of the tissue for prolonged retention; and/or exert cytotoxic effect on surrounding cells to release cellular content and trigger further inflammation.

In several pioneering studies, mBSA has been used as a designated carrier protein for DNA to immunize mice in order to induce anti-DNA antibodies [5]–[9]. It was recognized that the presence of this immunogenic carrier protein was a prerequisite for eliciting an immune response toward denatured DNA, as DNA is non-immunogenic [7]. Our data clearly show that mBSA directly binds to ssDNA, which prompts further conversion to amyloid, and the resulting mBSA-ssDNA complex elicits profound peritoneal inflammation in vivo.

Recently, we have shown that immunization of amyloid fibrils containing DNA can trigger non-autoimmune mice to develop lupus-like syndrome, including anti-DNA and anti-RNA and other autoantibodies [21]. The current molecular link between mBSA and amyloid-related proteins invites a closer look at the cellular and immunological functions of different misfolded protein species. While mBSA preferentially binds to denatured ssDNA, stabilized amyloid precursor protein we studied interacts readily with ssDNA and all natural nucleic acids including double-stranded DNA (dsDNA) and RNA. By dose titration, the cytotoxic activity of mBSA is rather repressed in comparison with stabilized amyloid precursor [20]. Inoculated together with DNA, both mBSA and stabilized amyloid precursor induced robust peritoneal infiltration in vivo. However, we were unable to detect the expression of type I interferon (IFN-I) after mBSA challenge (data not shown), whereas DNA-containing amyloid induced significant IFNα/β and interferon-inducible gene expression [21]. This is consistent with the result obtained with human primary pDCs: mBSA complexed with natural nucleic acids failed to induce IFNα/β (data not shown), a drastic contrast to nucleic acid-containing amyloid [21]. The consequence of lacking IFN-I may correlate with the limited antibody crossreactivity with autoantigens after mBSA-dsDNA immunization [4]. In contrary, DNA-containing amyloid provoked broad anti-self-antigen responses beyond the specific reactions to the immunogen, suggesting an essential role of type I interferon in autoimmunity.

By instigating strong inflammatory response, adjuvants are critical components of effective vaccine immunization. The finding that a seemingly simple protein mBSA may in fact act as an unintended adjuvant to induce various autoimmune or autoinflammatory disorders is intriguing. The current understanding about how immune tolerance is broken by environmental, exogenous or self-derived triggering factors remains incomplete. There are increasing evidence to suggest a potential association of vaccination and/or immunostimulatory adjuvants with autoimmune abnormalities. For instance, administration of tetanus toxoid vaccine may trigger antibodies targeting beta2-GPI, inducing Autoimmune/Autoinflammatory Syndrome Induced by Adjuvants (ASIA) [41], [42]. Injection of hydrocarbon oils such as pristane can cause chronic inflammation and induce systemic autoimmunity in non-lupus prone mice [43]. Therefore, the mechanism how molecules with strong adjuvant functions facilitate the development of autoimmunity deserves further in-depth investigation.

In conclusion, we have revealed an innate stimulatory adjuvant-like propensity of mBSA, which stems from its unique amyloid-like biochemical characteristics. This finding helps fully understand and properly interpret the phenotypes observed in different animal models.
